# PCR Primers for Metazoan Mitochondrial 12S Ribosomal DNA Sequences

**DOI:** 10.1371/journal.pone.0035887

**Published:** 2012-04-19

**Authors:** Ryuji J. Machida, Matthew Kweskin, Nancy Knowlton

**Affiliations:** National Museum of Natural History, Smithsonian Institution, Washington, D.C., United States of America; King Abdullah University of Science and Technology, Saudi Arabia

## Abstract

**Background:**

Assessment of the biodiversity of communities of small organisms is most readily done using PCR-based analysis of environmental samples consisting of mixtures of individuals. Known as metagenetics, this approach has transformed understanding of microbial communities and is beginning to be applied to metazoans as well. Unlike microbial studies, where analysis of the 16S ribosomal DNA sequence is standard, the best gene for metazoan metagenetics is less clear. In this study we designed a set of PCR primers for the mitochondrial 12S ribosomal DNA sequence based on 64 complete mitochondrial genomes and then tested their efficacy.

**Methodology/Principal Findings:**

A total of the 64 complete mitochondrial genome sequences representing all metazoan classes available in GenBank were downloaded using the NCBI Taxonomy Browser. Alignment of sequences was performed for the excised mitochondrial 12S ribosomal DNA sequences, and conserved regions were identified for all 64 mitochondrial genomes. These regions were used to design a primer pair that flanks a more variable region in the gene. Then all of the complete metazoan mitochondrial genomes available in NCBI's Organelle Genome Resources database were used to determine the percentage of taxa that would likely be amplified using these primers. Results suggest that these primers will amplify target sequences for many metazoans.

**Conclusions/Significance:**

Newly designed 12S ribosomal DNA primers have considerable potential for metazoan metagenetic analysis because of their ability to amplify sequences from many metazoans.

## Introduction

Human activities pose severe threats to planetary biodiversity, yet most marine species remain undescribed [1]–[3]. In this context, the ability to rapidly assess biodiversity at various spatio-temporal scales without assigning formal taxonomic names to all samples is urgently needed. Moreover, the species that comprise the majority of marine biodiversity are small and difficult to sample individually. Thus, the availability of second-generation sequencing methods has the potential to transform our ability to assess biodiversity via metagenomic and/or metagenetic approaches. Most studies to date have targeted microbes and protozoans [4]–[7] and only a limited number of studies have been carried out for metazoans [8], [9]. Unlike the situation with microbes, where analysis of the 16S ribosomal DNA sequence is standard, the appropriate gene for metazoan metagenetic studies is less clear. Although the cytochrome oxidase (COI) gene is routinely used for bar-coding [10]–[12], finding a single set of primers capable of amplifying most metazoans has been challenging. In the present study, we present data for a newly developed pair of primers that target the mitochondrial 12S ribosomal DNA sequence of many metazoans.

## Results

**Figure 1:**
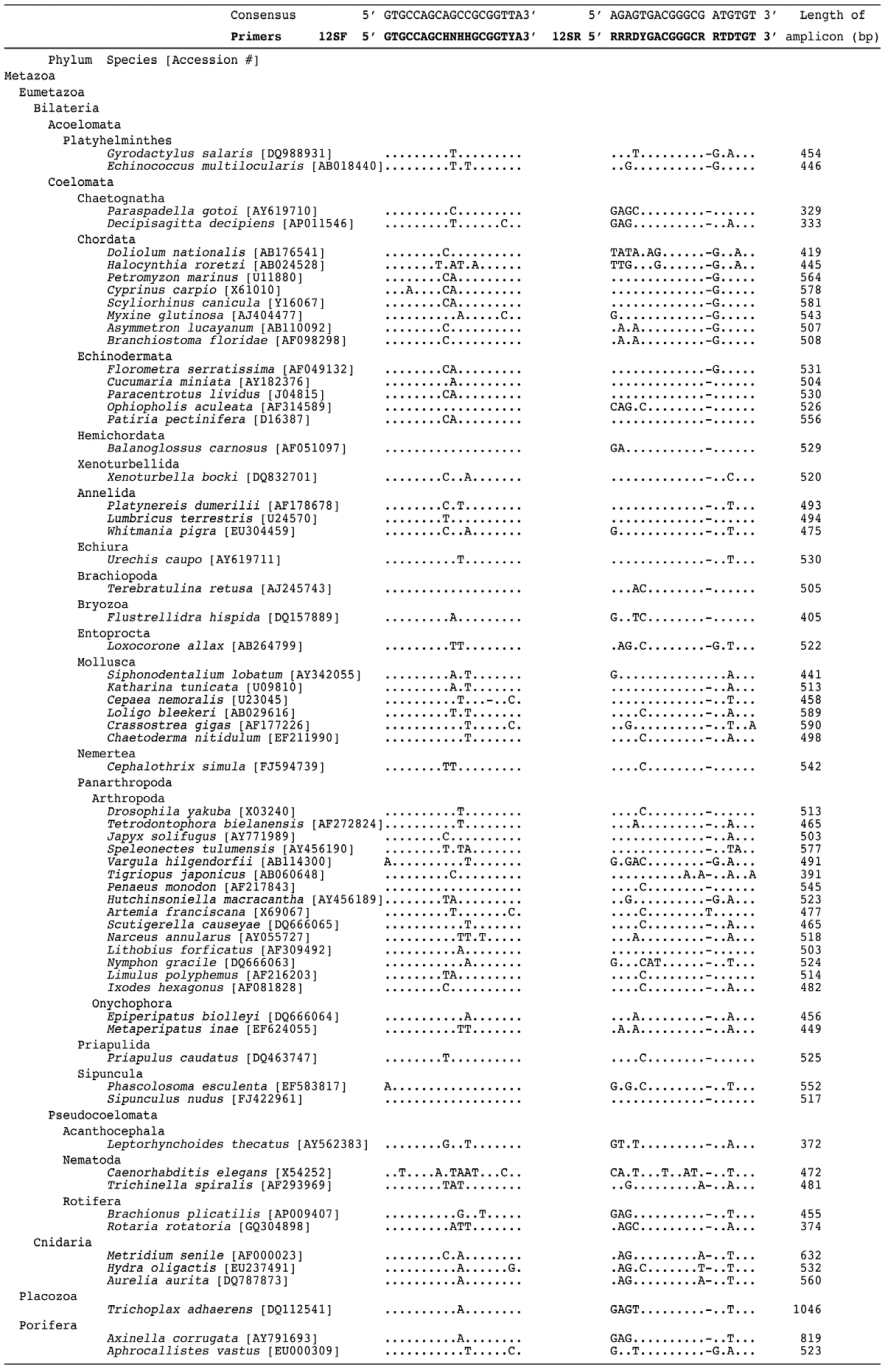
Sequence of primers and alignment of the conserved regions of 12S ribosomal DNA gene sequences of 64 metazoan species belonging to 23 phyla. Accession numbers of individuals are denoted in parentheses. Lengths of amplified PCR products without primers are indicated. Hierarchy of NCBI taxonomy database is followed in this figure.

We identified two conserved regions suitable for designing a pair of PCR primers by performing careful alignments of the 64 complete mitochondrial genomes chosen to represent all metazoan classes (Fig. 1). Lengths of expected PCR products using the primer pair were between 329 and 1046 bp, the majority of which (56 of 64) were 400–600 bp in length (Fig. 1).

To test likely efficacy of the primer pair, primer DNA sequences and the target regions for all available, complete metazoan mitochondrial genome sequences from NCBI were compared (Table 1). These sequences represent 23 phyla, but only eight phyla had ten or more sequences at the time of the analysis (Platyhelminthes, Chordata, Echinodermata, Mollusca, Arthropoda, Nematoda, Cnidaria, Porifera); the remainder typically had five or fewer sequences, so that the generality of the findings for these groups is more limited. We categorize the extent of primer compatibility by counting the number of mismatches between the forward and reverse primers and the downloaded metazoan mitochondrial genomes (no mismatches, one mismatch, two or more mismatches for both primers). Although many subtleties are missed in this characterization (e.g. just one mismatch on the 3′ end of the alignment will largely preclude amplification, whereas mismatches closer to the 5′ end will often have much lesser effects [13]), it provides some indication of groups where amplification problems are likely.

**Table 1:** Observed mismatches between the primer pair and the targeted region for all complete mitochondrial genome sequences downloaded from the NCBI Organelle Genome Resources database.

Phylum (# species)						
	No mismatch		One mismatch		Two or more mismatches	
	% (# species)		% (# species)		% (# species)	
	12SF	12SR	12SF	12SR	12SF	12SR
Metazoa						
Eumetazoa						
Bilateria						
Acoelomata						
Platyhelminthes (35)	91.43 (32)	68.57 (24)	8.57 (3)	28.57 (10)	0.00 (0)	2.86 (1)
Coelomata						
Deuterostomia						
Chaetognatha (5)	60.00 (3)	20.00 (1)	40.00 (2)	40.00 (2)	0.00 (0)	40.00 (2)
Chordata (1499)	90.99 (1364)	98.07 (1470)	7.94 (119)	0.80 (12)	1.07 (16)	1.13 (17)
Echinodermata (25)	92.00 (23)	76.00 (19)	8.00 (2)	24.00 (6)	0.00 (0)	0.00 (0)
Hemichordata (3)	66.67 (2)	100.00 (3)	33.33 (1)	0.00 (0)	0.00 (0)	0.00 (0)
Xenoturbellida (1)	100.00 (1)	0.00 (0)	0.00 (0)	100.00 (1)	0.00 (0)	0.00 (0)
Protostomia						
Annelida (9)	100.00 (9)	100.00 (9)	0.00 (0)	0.00 (0)	0.00 (0)	0.00 (0)
Echiura (2)	100.00 (2)	100.00 (2)	0.00 (0)	0.00 (0)	0.00 (0)	0.00 (0)
Brachiopoda (3)	100.00 (3)	100.00 (3)	0.00 (0)	0.00 (0)	0.00 (0)	0.00 (0)
Bryozoa (3)	100.00 (3)	100.00 (3)	0.00 (0)	0.00 (0)	0.00 (0)	0.00 (0)
Entoprocta (2)	100.00 (2)	100.00 (2)	0.00 (0)	0.00 (0)	0.00 (0)	0.00 (0)
Mollusca (103)	92.23 (95)	72.81 (75)	5.83 (6)	21.36 (22)	1.94 (2)	5.83 (6)
Nemertea (3)	100.00 (3)	100.00 (3)	0.00 (0)	0.00 (0)	0.00 (0)	0.00 (0)
Panarthropoda						
Arthropoda (354)	92.09 (326)	92.66 (328)	5.08 (18)	4.52 (16)	2.82 (10)	2.82 (10)
Onychophora (3)	100.00 (3)	100.00 (3)	0.00 (0)	0.00 (0)	0.00 (0)	0.00 (0)
Priapulida (1)	100.00 (1)	100.00 (1)	0.00 (0)	0.00 (0)	0.00 (0)	0.00 (0)
Sipuncula (2)	50.00 (1)	100.00 (2)	50.00 (1)	0.00 (0)	0.00 (0)	0.00 (0)
Pseudocoelomata						
Acanthocephala (1)	0.00 (0)	0.00 (0)	100.00 (1)	100.00 (1)	0.00 (0)	0.00 (0)
Nematoda (48)	4.17 (2)	4.17 (2)	2.08 (1)	8.33 (4)	93.75 (45)	87.50 (42)
Rotifera (2)	0.00 (0)	50.00 (1)	0.00 (0)	50.00 (1)	100.00 (2)	0.00 (0)
Cnidaria (33)	24.24 (8)	60.60 (20)	72.73 (24)	18.18 (6)	3.03 (1)	21.21 (7)
Placozoa (4)	100.00 (4)	100.00 (4)	0.00 (0)	0.00 (0)	0.00 (0)	0.00 (0)
Porifera (42)	88.10 (37)	97.62 (41)	9.52 (4)	2.38 (1)	2.38 (1)	0.00 (0)

Comparisons were performed for each phylum. Hierarchy of the NCBI taxonomy database is followed in this table.

For 11 phyla, 90% or more of the species with complete mitochondrial genomes in the dataset have no mismatches for both the forward and reverse primers. Of these, three are reasonably well sampled: the Chordata (1499 sequences), the Annelida (nine sequences), and the Arthropoda (354 sequences). For an additional eight phyla, more than 90% of species showed no more than one mismatch for the forward and/or reverse primers, including four phyla with 25 or more sequences in the dataset (Platyhelminthes, Echinodermata, Mollusca, Porifera). However, for one of these phyla, the Mollusca, most of the mismatches were concentrated in a single class, the Bivalvia (51% of species with one mismatch and 15% with two or more mismatches for the reverse primer, data not shown). Four phyla had two or more mismatches for the forward and/or reverse primers, and two of these, the Nematoda (48 sequences) and the Cnidaria (33 sequences), were well sampled. An especially high percentage of mismatches was observed for the megadiverse taxon Nematoda (94% and 88% of species with 2 or more mismatches for the forward and reverse primers, respectively; Table 1).

**Figure 2:**
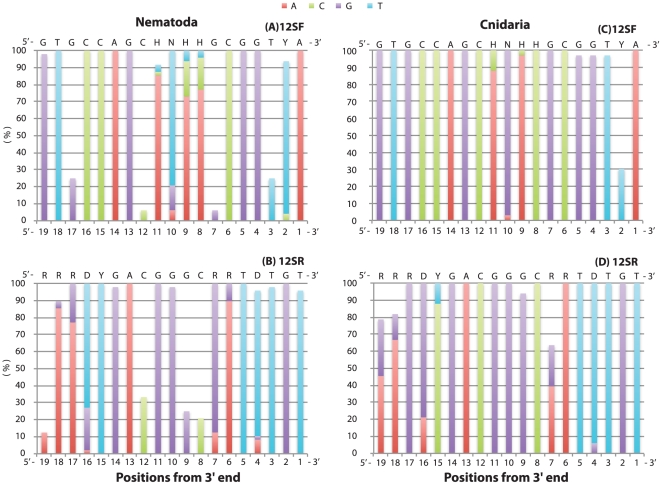
Nucleotide composition of the primer target regions observed in Nematoda (A and B) and Cnidaria (C and D). Sequences of the primers are indicated on top of each figure. The nucleotides that matched with the primers are shown stacked in each column. Position of each site from 3′ to 5′ is indicated on bottom from left to right.

Because the position of mismatches is known to influence amplification efficacy, with more serious amplification problems occurring when mismatches are close to the 3′ end [13], we examined this for the Nematoda and Cnidaria (Fig. 2). In the Nematoda, the 3rd, 7th, 12th, and 17th positions from 3′ end of the primer 12SF, and the 8th, 9th, 12th, and 19th positions from 3′ end of the primer 12SR, had low percentages of matches (<35%). Among the Cnidaria, the only position with a comparably low percentage of matches was the 2^nd^ position from 3′ end of the 12SF primer.

**Figure 3:**
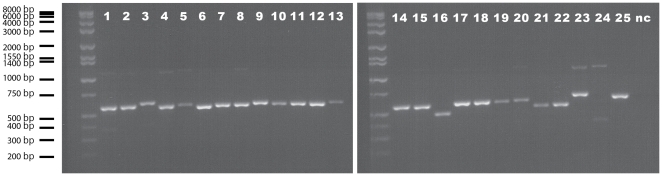
Agarose gel images of the compatibility test using PCR. The newly designed primers were used in PCR reactions for individuals belonging to various groups within six phyla: Sipuncula- 1 *Phascolosoma* sp.; Echinodermata- 2 *Ophiocoma erinaceus* (brittlestar); Chordata- 3 *Pseudamiops gracilicauda* (fish); Annelida (Polychaeta)- 4 *Pherecardia striata*, 5 unidentified terebellid species; Arthropoda- 6 *Xanthias latifrons* (brachyuran crab), 7 *Pilodius flavus* (brachyuran crab), 8 *Liomera* sp. (brachyuran crab), 9 *Carupa* sp. (brachyuran crab), 10 unidentified pilumnid species (brachyuran crab), 11 unidentified xanthid species (brachyuran crab), 12 *Calcinus gouti* (anomuran crab), 13 *Synalpheus* sp. (caridean shrimp), 14 *Periclimenes* sp. (caridean shrimp), 15 unidentified caridean shrimp, 16 unidentified amphipod species; Mollusca- 17 *Cypraea helvola* (gastropod), 18 *Cypraea fimbriata* (gastropod), 19 *Trivia* sp. (gastropod), 20 *Erato sandwichensis* (gastropod), 21 unidentified haminoeid species (gastropod), 22 *Berthellina* sp. (gastropod), 23 *Chlamys* sp. (bivalve), 24 *Lima* sp. (bivalve), 25 unidentified lucinid species (bivalve). nc-negative control. Good amplifications were observed for all individuals except one bivalve (24).

Finally, to test the primer pairs directly, PCR was performed for 25 animals belonging to six phyla (Sipuncula, Echinodermata, Chordata, Annelida, Arthropoda, Mollusca) (Fig. 3). Reliable PCR amplifications were obtained from all of these except for one bivalve species. To confirm the identity of the amplified products, bands with the expected length were cut out from the gel and sequenced for the first eight individuals in Fig. 3. Clear electropherograms for the 12S gene were obtained in all cases (data not shown).

## Discussion

Four genes represent good candidates for use in metazoan metagenetic analyses: the mitochondrial COI and 12S ribosomal DNA regions and the nuclear 18S and 28S ribosomal DNA regions. One factor that influences the choice of a target gene is the number of available sequences in public databases. The mitochondrial COI gene currently has advantages over the 12S gene in this regard. However, because of very rapid advances in sequencing technologies, more 12S sequences in databases of the future are anticipated.

The mode of evolution of these four genes is quite different. The nuclear 18S and 28S ribosomal DNA regions have slower evolutionary rates compared to the two mitochondrial genes [14]–[17]. Therefore, it is rather easy to design metazoan universal primers for the two nuclear genes, but their ability to discriminate closely related species is lower. The rate of evolution of the mitochondrial 12S ribosomal DNA region is generally much faster than that of the nuclear-encoded ribosomal genes, although it is slower than that of the mitochondrial COI gene [17]–[19]. Therefore, the 12S gene has the potential to discriminate congeneric taxa [20], [21], including some that are recently diverged (*Neocalanus plumchrus* and *N. flemingeri* [17], *Triconia minuta*, *T. umerus* and *T.* sp. 8 [19]; *Oncaea ovalis* and *O. parabathyalis* [19]), without sacrificing the ability to amplify species from a broad array of groups.

In this study, we identified two conserved regions in the 12S gene suitable for designing a pair of PCR primers. Based on an analysis of mismatches (Table 1), these primers are expected to succeed in amplifying 12S genes for many metazoans. Success of PCR is determined by multiple factors (e.g. annealing temperature, salt concentration, gDNA concentration, and contamination by inhibitors), but characteristics of the primers (specificity, length, melting temperature, GC content) and compatibility of the primers and target region sequences are the most important factors influencing success. In general, success of PCR can be expected even if there are a few mismatches between the sequences of the primer and the target region. If there is a species with better compatibility in the environmentally extracted gDNA, however, then this species will be amplified preferentially. Furthermore, target region sequences with mismatches located in the 5′ portion will be more effectively amplified than those with mismatches located in the 3′ portion [13]. Therefore, it is expected that groups with higher number of mismatches, especially in the 3′ portion of the primers, will be less effectively amplified when the primers are used for metagenetic analysis.

The groups most likely to be affected in this regard are the Bivalvia, Cnidaria and Nematoda. Given the diversity, ubiquity and small size of nematodes, it might be advisable to use specifically designed primers that target nematodes when performing analyses of metazoans from environmental samples. In addition, we are now preparing primer sets targeting metazoan nuclear 18S and 28S ribosomal DNA sequences. Those gene sequences have less capacity to discriminate closely related taxa, but have the ability to recover most metazoans (Machida and Knowlton, submit).

An additional concern is the possibility of amplification of bacterial DNA. To assess the extent of this possible source of error, DNA was extracted from 30 whole individuals, including gut contents and exoskeletons, representing six phyla (Sipuncula, Echinodermata, Chordata, Annelida, Arthropoda, and Mollusca). Preliminary Roche 454 sequence data reveal that there were some bacterial 16S ribosomal DNA sequences in the dataset, but more than 83% of quality-filtered (Mothur standard operating procedure [22]) sequences were metazoan mitochondrial 12S sequences based on BLAST searches against GenBank's collection of non-redundant nucleotide sequences (Machida and Knowlton, unpublished data).

Although we have successfully gotten good quality mitochondrial 12S ribosomal DNA sequences from various phyla using the primer pair, double bands were observed in PCR products from some individuals (Fig. 3). One possible reason for this phenomenon is the high degeneracy of the primers. One method to minimize the probability of double bands is to use hot-start taq polymerase together with a touchdown PCR thermal profile. Also, excising target length PCR products from agarose gels is good way to get clear sequences from individuals with double-banded PCR products.

## Materials and Methods

### Designing of PCR primer pair for mitochondrial 12S ribosomal DNA

A total of 64 complete mitochondrial genome sequences were downloaded from GenBank (Fig. 1) from each taxonomic level “class” within the Metazoa using the NCBI Taxonomy Browser. When the dating of sequence submission was clear, the oldest record of the genome sequence within the class was selected.

First, the mitochondrial 12S ribosomal DNA regions were excised from the genomes. Next, careful alignment of the sequences was performed using MAFFT: L-INS-i [23], and four conserved regions were identified. Out of the four regions, two were long enough to be able to design the primer pair. In some species, the target region was not identified by the alignment of MAFFT. In those cases, target regions were searched for using the aligned primer regions as seeding sequences. ClustalX was used for the search [24], and additional manual alignments were performed by MacClade 4.0.8a [25]. Although the ClustalX is a global alignment program, it was adequate for the analyses. Accession numbers of all sequences used in this study are listed in Fig. 1 (in the present study, no new sequences were generated).

### Compatibility test using PCR

The newly designed pair of primers was tested for individuals belonging to various phyla (Fig. 3). Extractions of DNA were performed using DNeasy Blood & Tissue Kit (Qiagen) following the manufacturer's protocol. PCR was done in a 9700 thermal cycler (Applied Biosystems), and reactions were carried out with a 15 µl reaction volume containing 9.8 µl of sterile, distilled H_2_O, 1.5 µl of 10× 2 SA PCR buffer (Clontech), 1.2 µl of dNTP (2.5 mM each), 0.6 µl of each primer (5 µM), 0.3 µl of Advantage 2 DNA Polymerase Mix (Clontech), and 1.0 µl of the templates. A PCR mixture without template was also prepared as a negative control. Initial denaturation was carried out at 95°C for 10 min. This long denaturation is important when the PCR is performed using hot-start polymerase. Touchdown PCR was applied for the reaction: denaturation at 95°C for 10 s, annealing at 62°C for 30 s, and extension at 72°C for 60 s. Temperatures for the annealing were progressively decreased with advancing cycles (−1.0°C per cycle) from 62 to 46°C during the first 16 cycles and kept constant at 46°C during the subsequent 25 cycles. PCR products were electrophoresed on a 2.0% TBE agarose gel containing ethidium bromide and visualized using an ultraviolet transilluminator.

### Compatibility test using all metazoan mitochondrial genome sequences in the NCBI Organelle Genome Resources database

A total of 2201 complete metazoan mitochondrial genome sequences (all those available) were downloaded from the NCBI Organelle Genome Resources database in February 2011 (ftp://ftp.ncbi.nlm.nih.gov/genomes/MITOCHONDRIA/Metazoa/). The 12S ribosomal DNA regions were excised from the genomes. During the excising, we found several genomes with incorrect or incomplete annotation of the 12S ribosomal RNA gene. These sequences were removed from the dataset, resulting in 2183 mitochondrial 12S mitochondrial DNA sequences used for the analysis. Using this sequence dataset, target regions for the primers were searched for based on three criteria: 1) complete match, 2) one mismatch, 3) two or more mismatches (Table 1). For Nematoda and Cnidaria, the positional patterns of mismatches with the primer pairs were also analyzed (Fig. 2).

## References

[1] Costello MJ, Coll M, Danovaro R, Halpin P, Ojaveer H (2010). A census of marine biodiversity knowledge, resources, and future challenges.. Plos One.

[2] McIntyre AD (2010). Life in the world's oceans: Diversity, distribution, and abundance.

[3] Snelgrove PVR (2010). Discoveries of the census of marine life: Making ocean life count.

[4] Sogin ML, Morrison HG, Huber JA, Mark Welch D, Huse SM (2006). Microbial diversity in the deep sea and the underexplored “rare biosphere”.. Proc Natl Acad Sci USA.

[5] Committee on Metagenomics, National Research Council (2007). Challenges and functional applications: The new science of metagenomics: Revealing the secrets of our microbial planet.

[6] Amaral-Zettler LA, McCliment EA, Ducklow HW, Huse SM (2009). A method for studying protistan diversity using massively parallel sequencing of V9 hypervariable regions of small-subunit ribosomal RNA genes.. Plos One.

[7] Pawlowski J, Christen R, Lecroq B, Bachar D, Shahbazkia HR (2011). Eukaryotic richness in the abyss: Insights from pyrotag sequencing.. Plos One.

[8] Creer S, Fonseca VG, Porazinska DL, Giblin-Davis RM, Sung W (2010). Ultrasequencing of the meiofaunal biosphere: practice, pitfalls and promises.. Mol Ecol.

[9] Fonseca VG, Carvalho GR, Sung W, Johnson HF, Power DM (2010). Second-generation environmental sequencing unmasks marine metazoan biodiversity.. Nat Commun.

[10] Hebert PDN, Cywinska A, Ball SL, deWaard JR (2003a). Biological identifications through DNA barcodes.. Proc R Soc Lond B.

[11] Hebert PDN, Ratnasingham S, deWaard JR (2003b). Barcoding animal life: cytochrome c oxidase subunit 1 divergences among closely related species.. Proc R Soc Lond B.

[12] Schindel DE, Miller SE (2005). DNA barcoding a useful tool for taxonomists.. Nature.

[13] Bru D, Martin-Laurent F, Philippot L (2008). Quantification of the detrimental effect of a single primer-template mismatch by real-time PCR using the 16S rRNA gene as an example.. Appl Environ Microbiol.

[14] Hillis DM, Dixon MT (1991). Ribosomal DNA: Molecular evolution and phylogenetic inference.. Q Rev Biol.

[15] Gibson JF, Skevington JH, Kelso S (2010). Placement of Conopidae (Diptera) within Schizophora based on mtDNA and nrDNA gene regions.. Mol Phylogenet Evol.

[16] Makowsky R, Cox CL, Roelke C, Chippindale PT (2010). Analyzing the relationship between sequence divergence and nodal support using Bayesian phylogenetic analyses.. Mol Phylogenet Evol.

[17] Machida RJ, Tsuda A (2010). Dissimilarity of species and forms of planktonic *Neocalanus* copepods using mitochondrial COI, 12S, Nuclear ITS, and 28S gene sequences.. Plos One.

[18] Mueller RL (2006). Evolutionary rates, divergence date, and the performance of mitochondrial genes in Bayesian phylogenetic analysis.. Syst Biol.

[19] Böttger-Schnack R, Machida RJ (2011). Comparison of morphological and molecular traits for species identification and taxonomic grouping of oncaeid copepods.. Hydrobiologia.

[20] van der Kuyl AC, Ballasina DLP, Dekker JT, Maas J, Willemsen RE (2002). Phylogenetic relationships among the species of the genus *Testudo* (Testudines: Testudiniae) inferred from mitochondrial 12S rRNA gene sequences.. Mol Phylogenet Evol.

[21] Li J, Zhao GH, Zou FC, Mo XH, Yuan ZG, Ai L (2010). Combined mitochondrial 16S and 12S rDNA sequences: an effective genetic marker for inter-species phylogenetic analysis of zoonotic trematodes.. Parasitol Res.

[22] Schloss PD, Westcott SL, Ryabin T, Hall JR, Hartmann M (2009). Introducing mothur: open-source, platform-independent, community-supported software for describing and comparing microbial communities.. Appl Environ Microbiol.

[23] Katoh K, Toh H (2008). Recent developments in the MAFFT multiple sequence alignment program.. Brief Bioinform.

[24] Larkin MA, Blackshields G, Brown NP, Chenna R, McGettigan PA (2007). Clustal W and clustal X version 2.0.. Bioinformatics.

[25] Maddison DR, Maddison WP (2000). MacClade 4: Analysis of phylogeny and character evolution.

